# Role of Uterine Artery Doppler Study Between 11 and 14 Weeks as a Predictor of Preeclampsia

**DOI:** 10.7759/cureus.63591

**Published:** 2024-07-01

**Authors:** Karpagam RK, Karthik Krishna Ramakrishnan, Dhivya Gunasekaran, Arun Aram, Paarthipan Natarajan

**Affiliations:** 1 Department of Radiology, Saveetha Medical College and Hospital, Saveetha Institute of Medical and Technical Sciences, Saveetha University, Chennai, IND

**Keywords:** pregnancy induced hypertension (pih), gestational age, resistive index (ri), pulsatility index (pi), preeclampsia, uterine artery doppler

## Abstract

Introduction

Preeclampsia is a serious complication marked by antepartum hemorrhage, resulting in severe maternal and fetal complications. Predicting this condition using placental dysfunction assessments, such as uterine artery Doppler ultrasound, is challenging due to the placenta's evolving structural and biochemical characteristics throughout different stages of pregnancy.

Objectives

To determine the sensitivity, specificity, positive predictive value (PPV), and negative predictive value (NPV) of the uterine artery Doppler Pulsatility Index (PI) and Resistive Index (RI) in predicting preeclampsia. To compare the Doppler ultrasound measurements between normal pregnancies and those that develop preeclampsia. To assess the diagnostic accuracy of uterine artery Doppler ultrasound in predicting gestational hypertension in addition to preeclampsia.

Methodology

Conducted as a prospective study, 116 antenatal mothers with computed gestational ages and scan gestational ages between 11 and 14 weeks, and a previous history of preeclampsia were included. Subjects with chronic hypertension or multiple gestations were excluded. Participants underwent uterine artery Doppler screening, during which the PI and RI were measured upon obtaining three consecutive similar waveforms, and the mean PI of the left and right arteries was calculated. The outcomes of patients with normal pregnancies and those who developed preeclampsia were compared. Data were entered into Microsoft Excel (Microsoft® Corp., Redmond, WA, USA) and analyzed using IBM SPSS Statistics for Windows, Version 23 (Released 2015; IBM Corp., Armonk, NY, USA).

Results

The mean PI among participants was 1.75 (±0.38), with a range from 1 to 2.75. The mean RI was 0.58 (±0.08), ranging from 0.45 to 0.8. The cutoff for the mean PI in predicting preeclampsia was 2.27, which showed a sensitivity of 92.9%, specificity of 97.1%, PPV of 81.47%, NPV of 99.01%, and a diagnostic accuracy of 96.59% (area under the curve (AUC): 0.982). The cutoff for the mean RI for predicting preeclampsia was 0.695, with a sensitivity of 85.7%, specificity of 98%, PPV of 85.47%, NPV of 98.04%, and diagnostic accuracy of 96.52% (AUC: 0.965). In predicting gestational hypertension, the cutoff for the mean PI was 1.975, with a sensitivity of 80%, specificity of 82.9%, PPV of 17.41%, NPV of 98.92%, and diagnostic accuracy of 82.78% (AUC: 0.848). The cutoff for the mean RI in predicting gestational hypertension was 0.615, showing a sensitivity of 80%, specificity of 80.2%, PPV of 15.4%, NPV of 98.89%, and diagnostic accuracy of 80.19% (AUC: 0.767).

Conclusion

The research demonstrated that aberrant readings in uterine Doppler ultrasound, specifically in the PI and RI, possess strong overall validity in forecasting the occurrence of preeclampsia.

## Introduction

Pregnancy is a critical period for both maternal and fetal health, influenced by numerous external and internal factors. In India, a substantial proportion, approximately 20-30%, of pregnancies are categorized as high-risk, contributing significantly to perinatal morbidity and mortality rates. Hypertensive disorders during pregnancy, notably, account for a considerable portion of maternal morbidity (18.35%) and mortality (0.96%) nationwide [1,2]. A systematic review underscores the prevalence of hypertensive disorders during pregnancy in India at 9%, with preeclampsia affecting around 5.4% of pregnancies. These statistics underscore the significant health challenges posed by hypertensive conditions during pregnancy in the country.

Preeclampsia, characterized by hypertension and proteinuria, poses severe threats to both maternal and fetal well-being. Additionally, it can lead to complications such as fetal growth restriction, pulmonary edema, hemolysis, thrombocytopenia, liver dysfunction, and renal damage [3,4]. The condition manifests in two primary phenotypes: one characterized by low cardiac output and high vascular resistance, and the other by high cardiac output and low vascular resistance [5,6]. Early detection of preeclampsia is crucial for devising treatment plans and effectively monitoring therapy.

The global impact of preeclampsia on maternal and neonatal morbidity and mortality is profound. With timely and effective management, outcomes for affected women can be significantly improved. This emphasizes the importance of developing strategies for predicting and preventing preeclampsia and its complications, thereby ensuring optimal prenatal care [7,8].

Screening for preeclampsia involves considering various maternal factors, biomarkers, and ultrasound parameters. Guidelines from organizations such as the National Institute for Health and Care Excellence (NICE) and the Prediction and Prevention of Preeclampsia and Intrauterine Growth Restriction (PRECOG) stress the significance of assessing a woman's previous history of preeclampsia. Biomarkers like pregnancy-associated plasma protein-A (PAPP-A), placental protein 13 (PP-13), cystatin C, pentraxin 3, placental growth factor (PlGF), vascular endothelial growth factor (VEGF), inhibin A, and neutrophil gelatinase-associated lipocalin (NGAL) have been implicated in screening. Ultrasound parameters, particularly those indicating abnormal placentation such as increased resistance in uteroplacental circulation and the presence of diastolic notching in the uterine artery Doppler waveform, are also important.

Despite advancements in understanding the pathophysiology of hypertensive disorders during pregnancy, accurately identifying women at risk remains challenging. This limitation hampers the development and evaluation of preventive interventions. While various measurements of placental dysfunction have been linked to an increased risk of adverse pregnancy outcomes, evidence supporting their reliability in predicting these outcomes is limited. Additionally, the dynamic nature of placental structural and biochemical characteristics as gestational age progresses presents timing challenges for these measurements.

This study seeks to assess the utility of uterine artery Doppler ultrasound during the 11 to 14 weeks of gestation in predicting preeclampsia. By examining its effectiveness in identifying and managing this challenging condition early on, we aim to provide valuable contributions to prenatal care practices.

## Materials and methods

Based on the research conducted by Khanam et al. [9], which provided the specificity of the uterine artery Pulsatility Index (PI) color Doppler ultrasound as 98.9%, with a precision of 2.25% and a 95% confidence interval, the sample size was calculated using the formula: N = Z²×Sp×(1-Sp)​/d²(1-p).

Here, Z represents the z-value for a 95% confidence interval (1.96), Sp denotes specificity (0.989), d stands for precision (0.0225), and p indicates the prevalence of preeclampsia (0.05). This computation resulted in a sample size of 86. To account for potential non-responses, the sample size was increased by 15%, leading to a total required sample size of approximately 100 participants.

The research study titled "Role of Uterine Artery Doppler Study Between 11 and 14 Weeks as a Predictor of Preeclampsia" has been reviewed and approved by the Scientific Review Board (SRB) of Saveetha Medical College and Hospital, Chennai, India. The study protocol was evaluated to ensure it adheres to ethical guidelines and standards for research involving human subjects. Following a thorough review process, the SRB granted approval for the study to proceed. The official SRB approval number assigned to this research is 017/03/2024/PG/SRB/SMCH. This approval indicates that the study meets the necessary ethical requirements and safeguards for participant welfare and data integrity.

This prospective cohort study was conducted to evaluate the effectiveness of first-trimester uterine artery Doppler ultrasound in predicting preeclampsia. A total of 150 pregnant women were recruited from Saveetha Medical College and Hospital. Inclusion criteria included pregnant women with a gestational age between 11 and 14 weeks and a previous history of preeclampsia. Exclusion criteria included chronic hypertension and multiple gestations. Participants provided informed consent before enrollment. The Doppler ultrasounds were performed using the 2D GE Voluson (GE Healthcare, Chicago, IL, USA) and Mindray (Mindray, Mahwah, NJ, USA) ultrasound machines. Operators, who were trained postgraduates and senior residents, positioned the participants supine and obtained sagittal sections of the uterus to identify the cervical canal and internal os. Color flow mapping was used to locate each uterine artery adjacent to the cervix and uterus at the level of the internal os. The pulsed wave Doppler method was employed with a sampling gate set at 2 mm, ensuring the angle of insonation remained under 30 degrees. Three consecutive similar waveforms were obtained, and the mean PI and Resistive Index (RI) of the left and right arteries were calculated.

Upon obtaining three consistent waveforms, measurements of the PI and peak systolic velocity (PSV) were taken, followed by the calculation of the mean PI of the left and right arteries. The study further investigated pregnancy outcomes by contrasting those of patients with a normal pregnancy against those who developed preeclampsia. To determine the predictive accuracy of the PI, receiver-operating characteristic (ROC) curves were meticulously calculated.

**Figure 1:**
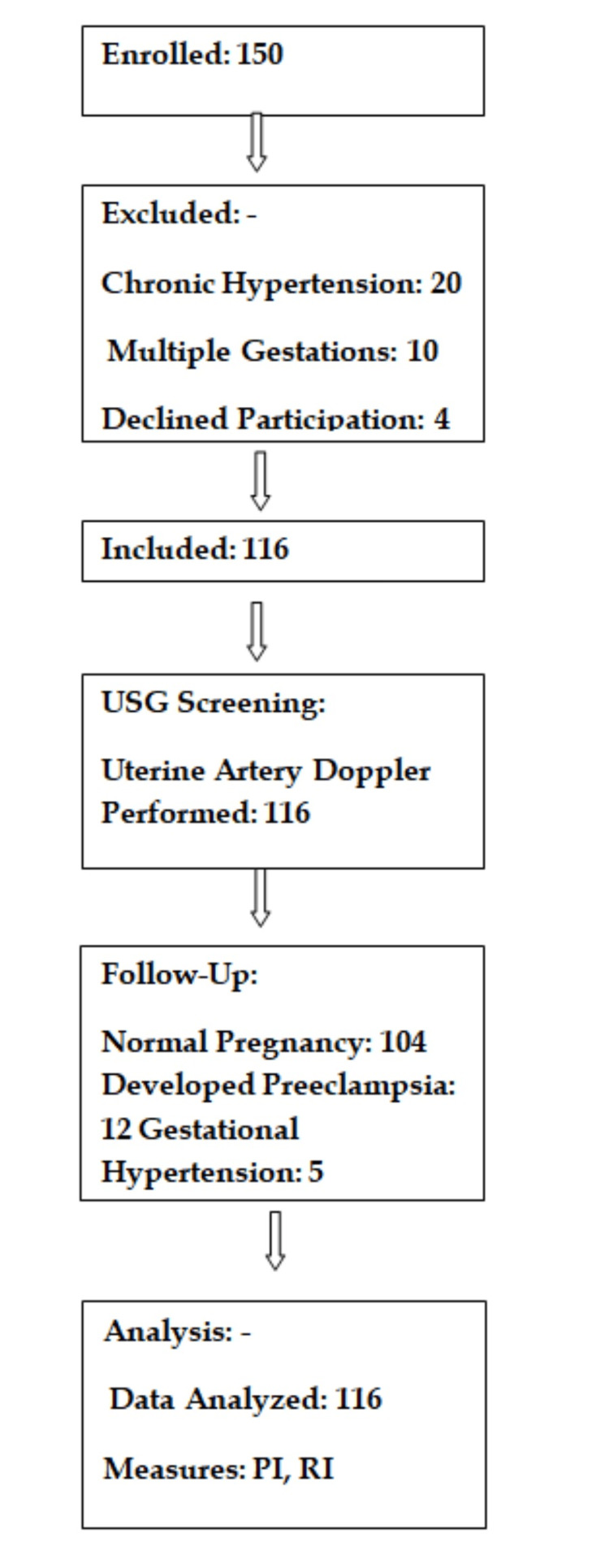
Flow diagram according to STARD 2015 guidelines STARD: Standards for reporting of diagnostic accuracy; PI: Pulsatility index; RI: Resistive index

In this study, we enrolled 150 participants and included 116 after applying exclusion criteria. The participant flow is detailed as per Standards for Reporting of Diagnostic Accuracy (STARD) 2015 guidelines in (Figure 1).

Regarding statistical methods, the study employed both descriptive and inferential statistics. Numerical variables, such as age, PI, RI, etc., were represented using mean, standard deviation, median, and mode, with histograms utilized for visual representation where necessary. Categorical variables, including gender and reported history of previous preeclampsia, were displayed as frequencies and percentages.

Data entry was carried out, followed by analysis with IBM SPSS Statistics for Windows, Version 23 (Released 2015; IBM Corp., Armonk, NY, USA). For inferential statistics, when a categorical variable, such as gestational age at 11-13 weeks, was compared with a continuous variable such as the PI and RI, data were visually presented through tables and bar diagrams. Significance testing was conducted using the one-way analysis of variance (ANOVA) test.

Additionally, ROC analysis was performed to predict outcomes related to gestational hypertension and pre-eclampsia with respect to PI and RI. Binomial logistic regression analysis was also conducted to evaluate the probability of pre-eclampsia based on PI and RI, considering p-values less than 0.05 as statistically significant.

## Results

**Figure 2:**
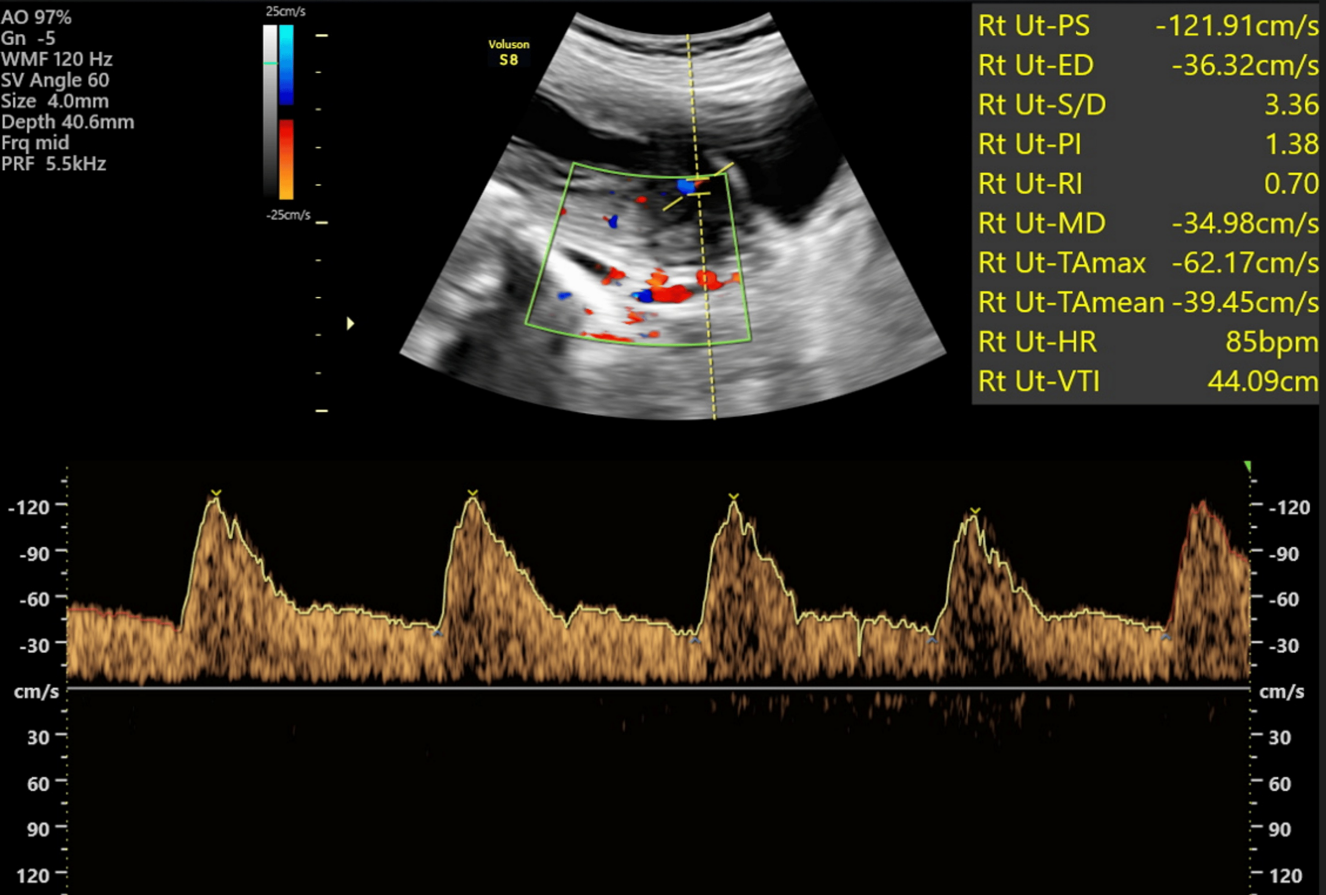
A primigravida exhibiting a Doppler flow pattern with normal resistance

**Figure 3:**
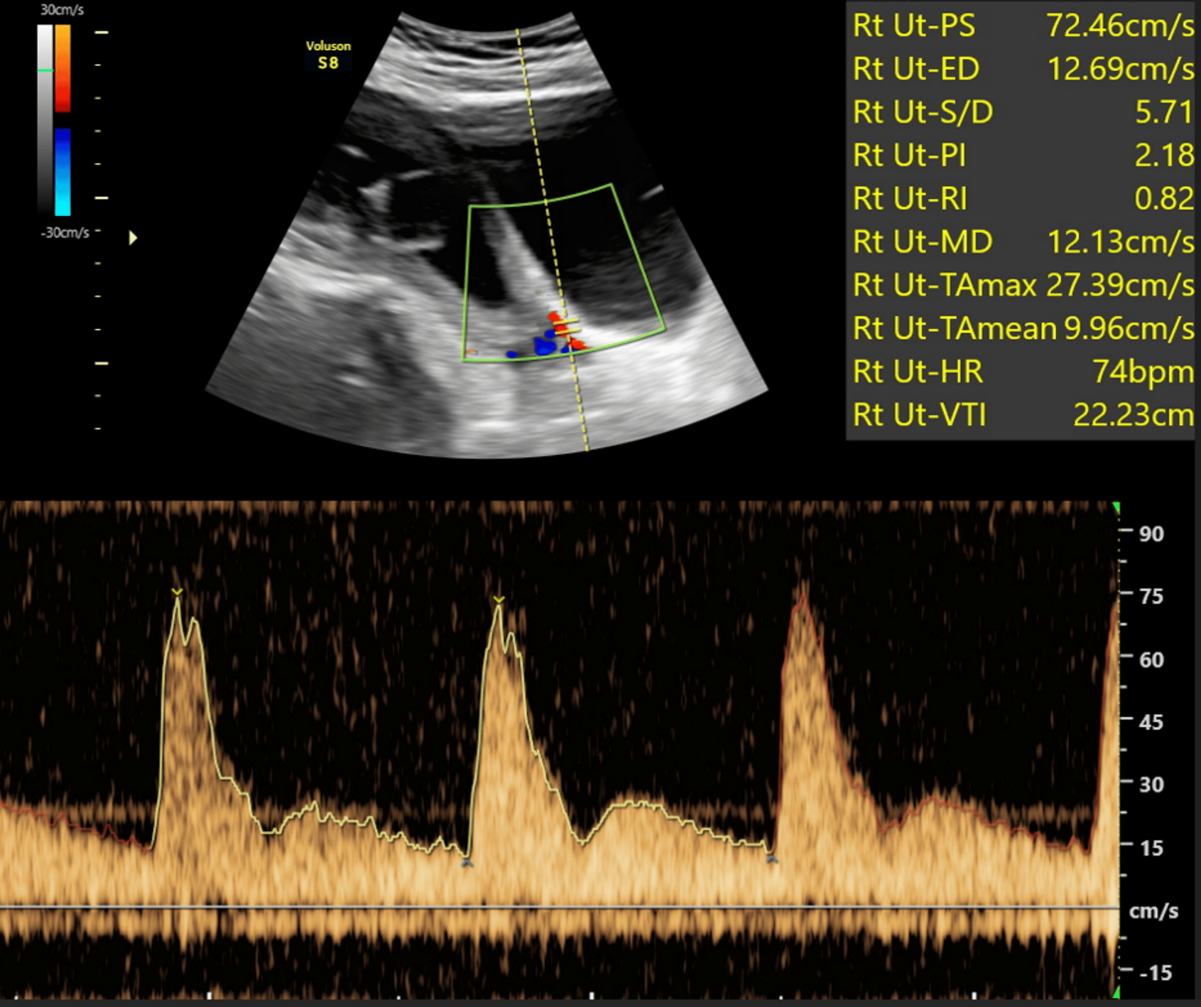
During the 12th week of pregnancy, a primigravida displayed high resistance flow on Doppler, later leading to the development of gestational hypertension during follow-up at 32 weeks

**Figure 4:**
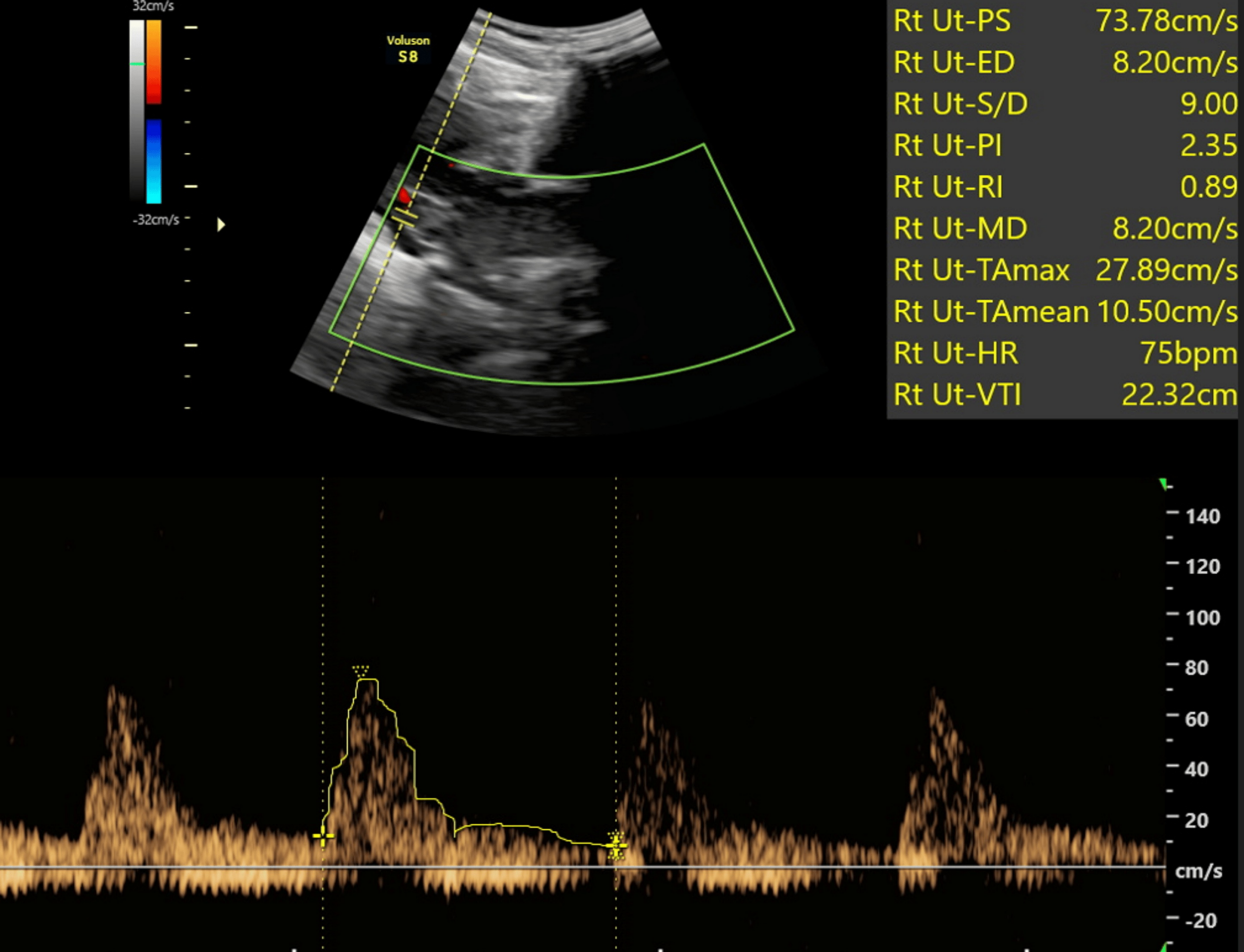
The uterine Doppler study conducted on a G2 P1L1 mother at 12 weeks revealed high resistance flow, characterized by a resistive index (RI) of 0.89 and pulsatility index (PI) of 2.35 Subsequently, the patient developed preeclampsia at 34 weeks during follow-up

**Figure 5:**
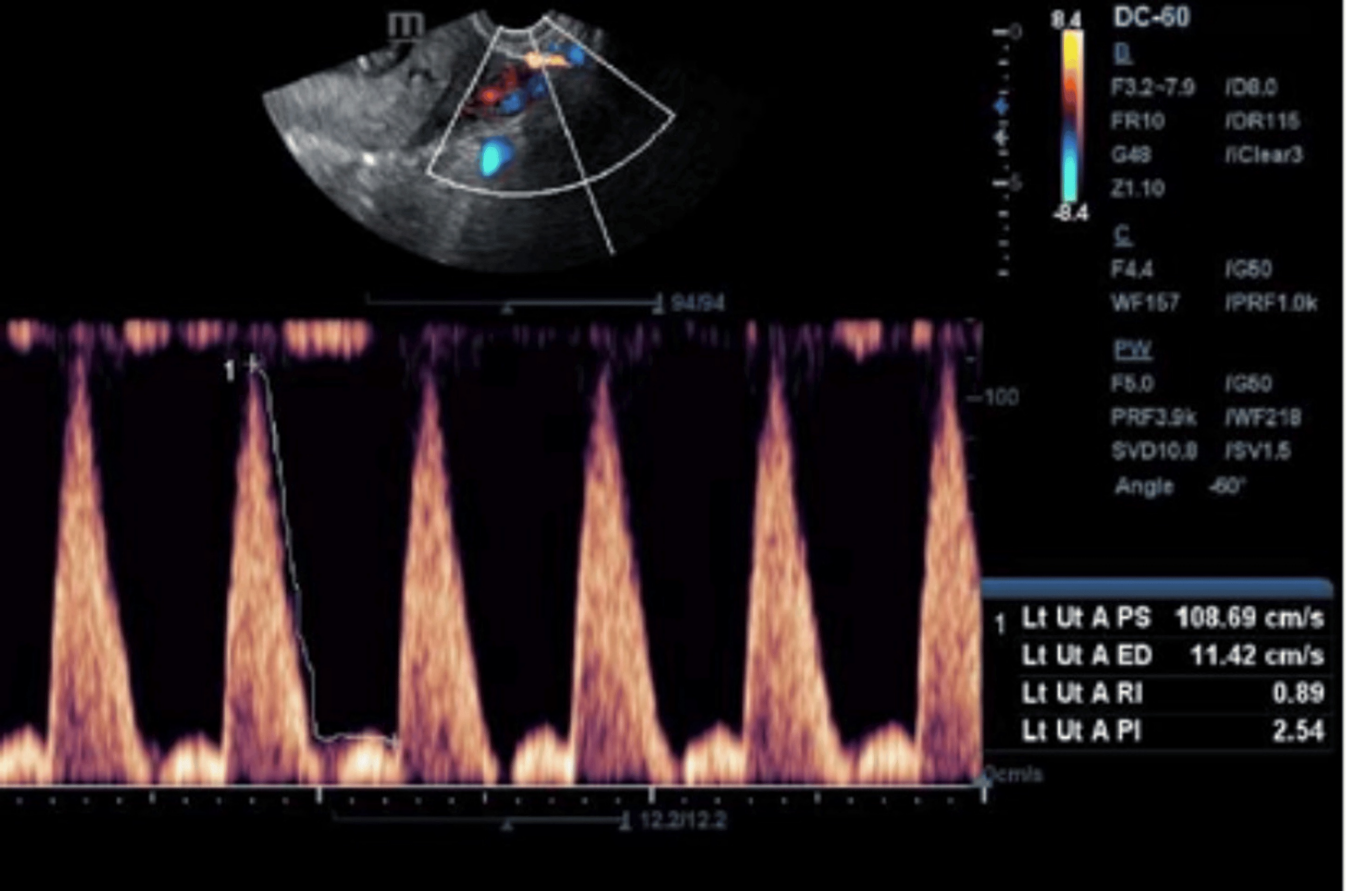
A primigravida displaying high resistance flow on uterine artery Doppler, characterized by an early diastolic notch with a resistivity index (RI) of 0.9 and pulsatility index (PI) of 2.54 During follow-up, the patient was diagnosed with preeclampsia at 30 weeks

Representative images are shown: Figure 2 presents a standard uterine artery Doppler study revealing normal flow patterns, while Figure 3 depicts a case of a first-time pregnant woman displaying high-resistance flow in Doppler, later developing gestational hypertension at 32 weeks. Figure 4 showcases a uterine Doppler study of a woman who has had one child previously, conducted at 12 weeks, exhibiting increased resistance flow with an RI of 0.86 and PI of 2.5; subsequent monitoring revealed the onset of preeclampsia at 34 weeks. Finally, Figure 5 illustrates a primigravida with elevated resistance flow on uterine artery Doppler, featuring an early diastolic notch with an RI of 0.9 and PI of 2.54; preeclampsia was diagnosed during follow-up at 30 weeks. 

The study conducted a thorough analysis of various parameters, as presented in Table 1, to evaluate the efficacy of first-trimester screening using uterine artery Doppler in predicting preeclampsia and gestational hypertension. These results were systematically organized under several headings, encompassing demographic details, clinical history, ultrasound measurements, and outcomes of statistical analysis.

**Table 1:** Parameters and comparison PI: Pulsatility index; RI: Resistivity index; AUC: Area under curve

Parameters	Description
Demographics	
Age	Average: 24.15 years; median: 24 years
Age group	21-25 years: 65.52%; 26-30 years: 27.59%; ≤20 years: 4.31%
Basic measurements	
Gestational age (GA)	Average: 12.56 weeks; range: 11.14-13.86 weeks
Crown-rump length (CRL)	Average: 6.07 cm; range: 4.2-7.8 cm
Nuchal translucency (NT)	Average: 2.18 mm; range: 1.7-2.9 mm
Pregnancy history	
Parity	
Primi	55.17%
G2P1L1	42.24%
G3P1L1A1	1.72%
G3P2L2	0.86%
Health conditions	
Pregnancy-induced hypertension (PIH)	Present: 5.17%
Gestational diabetes mellitus (GDM)	Present: 7.76%
Ultrasound indices	
Pulsatility index (PI)	Right mean: 1.96; left mean: 1.74; range: 1.1-2.7
Resistive index (RI)	Right mean: 0.57; left mean: 0.58; range: 0.4-0.8
Pregnancy complications	
Preeclampsia	Occurrence: 12.07%
Gestational hypertension	Occurrence: 4.31%
Predictive analysis for preeclampsia	
Using PI	AUC: 0.982; cutoff: 2.27; sensitivity: 92.9%; specificity: 97.1%
Using RI	AUC: 0.965; cutoff: 0.695; sensitivity: 85.7%; specificity: 98.0%
Predictive analysis for gestational hypertension	
Using PI	AUC: 0.848; cutoff: 1.975; sensitivity: 80.0%; specificity: 82.9%
Using RI	AUC: 0.767; cutoff: 0.615; sensitivity: 80.0%; specificity: 80.2%
Logistic regression for predicting preeclampsia	
Mean PI	Odds ratio: 497.83 (p = 0.010)
Mean RI	Odds ratio: 3.563 x 10^6 (p = 0.079)
Constant	p = 0.001

Participant ages exhibited variation, with the study categorizing them into different age groups to explore any potential correlation with the occurrence of preeclampsia or gestational hypertension. Additionally, the parity, or number of previous births, was documented to provide insights into its potential influence on pregnancy outcomes. Gestational age, quantified both in weeks and grouped into categories, along with measurements such as crown-rump length (CRL) and nuchal translucency (NT), was meticulously recorded for its relevance in early pregnancy assessment.

The historical records regarding pregnancy-induced hypertension (PIH) and gestational diabetes mellitus (GDM) were of paramount importance due to their known associations with unfavorable pregnancy outcomes. These variables underwent thorough examination alongside Doppler ultrasound measurements, particularly emphasizing the significance of the PI and RI. These indices are pivotal in assessing uteroplacental blood flow and anticipating complications such as preeclampsia and gestational hypertension.

The study further investigated the relationship between mean PI and mean RI with gestational age, elucidating how these indices vary throughout different stages of pregnancy and their predictive value for hypertensive disorders. ROC curves were meticulously analyzed to predict preeclampsia using PI and RI, as well as to predict gestational hypertension, thereby highlighting the diagnostic accuracy of these indices in early pregnancy.

Moreover, binomial logistic regression analysis was employed to refine the predictive model for preeclampsia, recognizing the multifactorial nature of this condition. This comprehensive analytical approach facilitated a nuanced understanding of how various factors interact in the risk of developing preeclampsia and gestational hypertension, underscoring the utility of first-trimester uterine artery Doppler screening in identifying at-risk pregnancies. By thoroughly collecting and analyzing data from diverse categories outlined in Table 1, the study offers valuable insights into the early identification and potential management strategies for hypertensive disorders during pregnancy.

Table 2 summarizes the diagnostic efficacy of mean PI and mean RI for predicting preeclampsia and gestational hypertension. It includes metrics such as sensitivity, specificity, positive predictive value (PPV), negative predictive value (NPV), and diagnostic accuracy, alongside the AUC values. For predicting preeclampsia, the mean PI and RI showed high diagnostic accuracy, sensitivity, and specificity, with AUCs of 0.982 and 0.965, respectively. In predicting gestational hypertension, both indices demonstrated good diagnostic potential, though with slightly lower AUC values of 0.848 for PI and 0.767 for RI. This concise representation provides a clear overview of the indices' performance in prenatal screening.

**Table 2:** Diagnostic performance of mean PI and mean RI in predicting preeclampsia and gestational hypertension PI: Pulsatility index; RI: Resistive index; HTN: hypertension

Test result variable	Area under the curve	Cut off	Sensitivity	Specificity	PPV	NPV	Diagnostic accuracy
Mean PI in predicting preeclampsia	0.982 (0.959-1)	2.27	92.90%	97.10%	81.47%	99.01%	96.59%
Mean RI in predicting preeclampsia	0.965 (0.922-1)	0.695	85.70%	98%	85.47%	98.04%	96.52%
Mean PI in predicting gestational HTN	0.848 (0.76-0.936)	1.975	80%	82.90%	17.41%	98.92%	82.78%
Mean RI in predicting gestational HTN	0.767 (0.527-1)	0.615	80%	80.20%	15.40%	98.89%	80.19%

## Discussion

The principal aim of our study was to evaluate the efficiency of first-trimester screening using uterine artery Doppler sonography, particularly in predicting preeclampsia by incorporating clinical and biochemical parameters. Through comprehensive analysis, our findings highlight the pivotal role of PI and RI in early detection, marking a significant step forward in prenatal care [10-13].

In our cohort, the mean PI values for the right and left uterine arteries were 1.96 (±0.37) and 1.74 (±0.4), respectively, with an overall mean PI of 1.75 (±0.38). Correspondingly, the mean RI values for both the right and left uterine arteries were 0.58, with a slight variance in standard deviation. The PI values demonstrated a notable decrease from weeks 11 through 13 of gestation, with this decrease reaching statistical significance (p < 0.05). In contrast, RI values showed insignificant fluctuations during the same period (p > 0.05).

Our study's findings align with previous research, such as that conducted by Gomez et al. [14], which also reported consistent PI measures across similar gestational weeks. This parallel underscores the reliability of PI as a consistent marker in early pregnancy assessments. The delineation of cutoff points for PI and RI in predicting preeclampsia not only underscores diagnostic precision but also highlights the predictive value and diagnostic accuracy of these indices. The sensitivity, specificity, PPV, NPV, and overall diagnostic accuracy figures stand as a testament to the robustness of PI and RI in early detection protocols [15].

Moreover, our investigation into the cutoff values for PI and RI concerning gestational hypertension reveals significant diagnostic capabilities. However, there is a marked disparity in PPVs between preeclampsia and gestational hypertension. This discrepancy highlights the subtle nature of these indices in differentiating between gestational hypertension and preeclampsia, underscoring the critical need for tailored screening protocols. Comparative analysis with previous studies, such as those by Khanam et al. [9], reveals higher sensitivity in our study for detecting preeclampsia using mean PI. This discrepancy points to the evolving understanding and application of these indices in prenatal screening. Furthermore, comparisons with studies by Oancea et al. and Abdel Moety et al. [16,17] elucidate variations in specificity and sensitivity rates, highlighting diversified outcomes stemming from different research methodologies and demographic considerations.

The studies conducted by Khanam et al., as well as Shahid et al. [9,18], provide a contextual backdrop for our findings. The ROC AUC values reported in these studies underscore the predictive accuracy of uterine artery PI in the screening for gestational hypertension and preeclampsia. The observed divergence in sensitivity and specificity metrics, particularly when compared to Shahid et al.’s [18] findings, highlights the evolving landscape of prenatal screening research. Our study’s consistency with these broader research trends reaffirms the critical role of uterine artery Doppler indices in early pregnancy screening.

The comprehensive analysis, underscored by comparative evaluations, establishes the uterine artery Doppler’s PI as a paramount marker in the screening for gestational hypertension and preeclampsia. This affirmation not only validates the efficacy of first-trimester screening protocols but also emphasizes the need for ongoing research and refinement in prenatal care practices.

Our study contributes to the growing body of evidence supporting the use of uterine artery Doppler sonography in early pregnancy screening and underscores the subtle differences in predicting preeclampsia and gestational hypertension. The highlighted sensitivity, specificity, PPV, NPV, and diagnostic accuracy across PI and RI indices serve as a guide for future research directions. This necessitates a collaborative strategy that combines clinical, biochemical, and sonographic parameters to improve prenatal care and outcomes. As we navigate through the complexities of early pregnancy screening, the insights derived from this study illuminate the path toward optimized maternal and fetal health.

This study demonstrated several significant strengths. One of the primary strengths is the use of first-trimester uterine artery Doppler ultrasound for the early detection of preeclampsia and gestational hypertension. The high predictive accuracy of the PI and RI is notable, supported by robust statistical measures such as sensitivity, specificity, and AUC values. The identification of specific cutoff values for PI and RI adds a novel contribution to the existing body of research, offering valuable reference points for early diagnosis and intervention. These findings have important clinical implications, suggesting that incorporating these Doppler ultrasound measurements into routine prenatal care could improve outcomes for at-risk pregnancies by enabling earlier detection and management of potential complications.

However, there are several limitations to this study. The study was conducted at a single center, which may limit the generalizability of the findings to other populations and clinical settings. Additionally, while the sample size of 150 participants provided valuable data, a larger sample size could enhance the robustness and reliability of the results. Another limitation is the exclusion of notching assessment in the Doppler ultrasound evaluations, which could potentially overlook certain high-risk individuals. Finally, the lack of longitudinal data, including perinatal and postnatal follow-up, restricts our ability to assess the long-term implications and predictive reliability of the screening method. These limitations highlight areas for future research to further validate and expand upon these findings.

Implication, including using uterine artery Doppler ultrasound in the first trimester, can improve prenatal care by enabling early detection of preeclampsia and gestational hypertension. Early detection allows for timely interventions, improving pregnancy outcomes for at-risk women. Incorporating this screening tool into routine prenatal care protocols could lead to more personalized and effective management strategies, reducing the incidence of adverse outcomes.

Future research should focus on validating these findings in larger, more diverse populations. Studies with longitudinal data to track perinatal and postnatal outcomes will help understand the long-term benefits of early detection. Investigating the inclusion of notching assessment in Doppler evaluations could provide a more comprehensive risk assessment. These efforts will build on the current study and contribute to better screening and management protocols for preeclampsia and gestational hypertension.

## Conclusions

In this prospective study, we assessed 116 pregnant women between 11 and 14 weeks of gestation, including those with a history of preeclampsia. The primary objective was to evaluate the efficacy of first-trimester uterine artery Doppler screening in predicting preeclampsia by determining key parameters such as sensitivity, specificity, PPV, and NPV, focusing on clinical and biochemical factors. The screening procedure involved measuring the PI and PSV from uterine artery Doppler waveforms, followed by calculating the mean PI for both left and right arteries. This methodology facilitated the comparison of pregnancy outcomes between normal pregnancies and those that later developed preeclampsia, with efficacy evaluated using ROC curves.

Our analysis revealed that the mean PI across the study cohort was 1.75 (±0.38), with values ranging from 1 to 2.75, and the mean RI was 0.58 (±0.08), ranging from 0.45 to 0.8. A PI cutoff of 2.27 exhibited high predictive accuracy for preeclampsia, with a sensitivity of 92.9%, specificity of 97.1%, PPV of 81.47%, NPV of 99.01%, and diagnostic accuracy of 96.59%. Similarly, an RI cutoff of 0.695 demonstrated a sensitivity of 85.7%, specificity of 98%, PPV of 85.47%, NPV of 98.04%, and diagnostic accuracy of 96.52% for predicting preeclampsia. For gestational hypertension, a PI cutoff of 1.975 and an RI cutoff of 0.615 yielded sensitivities of 80%, specificities of 82.9% and 80.2%, PPVs of 17.41% and 15.4%, NPVs of 98.92% and 98.89%, and diagnostic accuracies of 82.78% and 80.19%, respectively. In summary, our study underscores the potential of first-trimester uterine artery Doppler screening, particularly through PI and RI measurements, as highly effective predictive tools for both preeclampsia and gestational hypertension. These findings have significant implications for early detection and management strategies in pregnancies considered at risk, thereby making a substantial contribution to the advancement of prenatal care practices. The practical implications of these findings for clinical practice are discussed, particularly how using uterine artery Doppler in the first trimester can improve prenatal care and outcomes for at-risk pregnancies. We also suggest specific areas for future research based on our study’s findings and limitations, demonstrating a forward-looking approach and contextualizing our study within ongoing research efforts.
